# Supplementary material for: Structural variation, selection, and diversification of the NPIP gene family from the human pangenome
Source: Cell Genom. 2025 Aug 22;5(10):100977. doi: 10.1016/j.xgen.2025.100977 (PMC12790995; doi:10.1016/j.xgen.2025.100977)
Supplement: Document S1. Figures S1–S8 and Table S4 [file mmc1.pdf]

Cell Genomics, Volume *5*

Supplemental information

Structural variation, selection, and diversification of the *NPIP* gene family from the human pangenome

Philip C. Dishuck, Katherine M. Munson, Alexandra P. Lewis, Max L. Dougherty, Jason G. Underwood, William T. Harvey, PingHsun Hsieh, Tomi Pastinen, and Evan E. Eichler

SUPPLEMENTAL INFORMATION

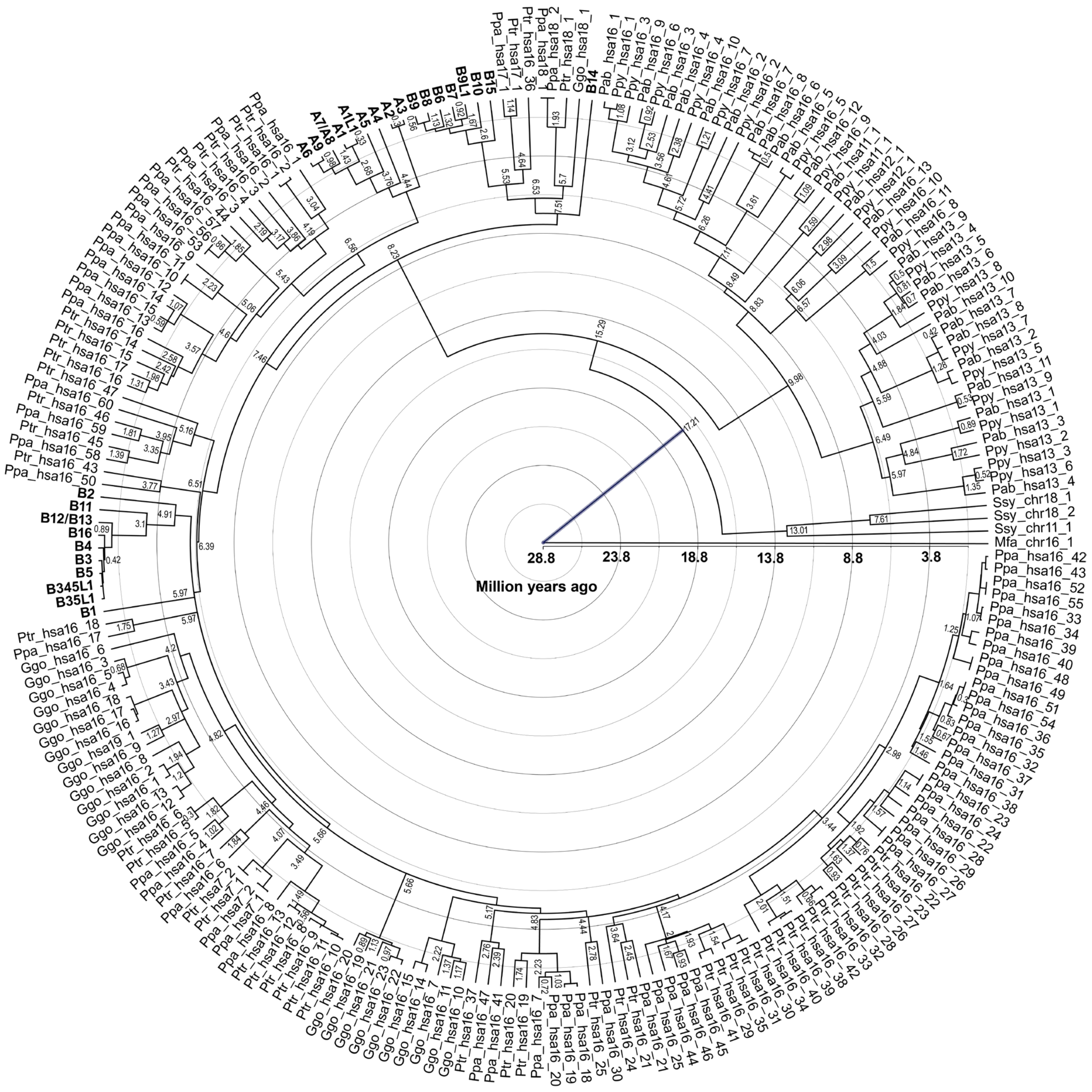

Figure S1. Timetree of human and ape *NPIP* duplications, related to Figure 2. The estimated age of *NPIP* paralogs for humans and ape species *Pan troglodytes* (Ptr), *Pan paniscus* (Ppa), *Gorilla gorilla* (Ggo), *Pongo pygmaeus* (Ppy), *Pongo abelii* (Pab), *Symphalangus syndactylus* (Ssy) is shown on a neutral phylogeny. The tree is rooted to the single copy ancestral *NPIP* from *Macaca fascicularis* (Mfa), with divergence time set to 28.8 mya. Human paralogs are bolded, and nonhuman primate paralogs are labeled by species abbreviation, chromosome number (hsa: human homologous chromosome, chr: species chromosome name), and position within the chromosome. Branch time estimates are indicated at the branch point.

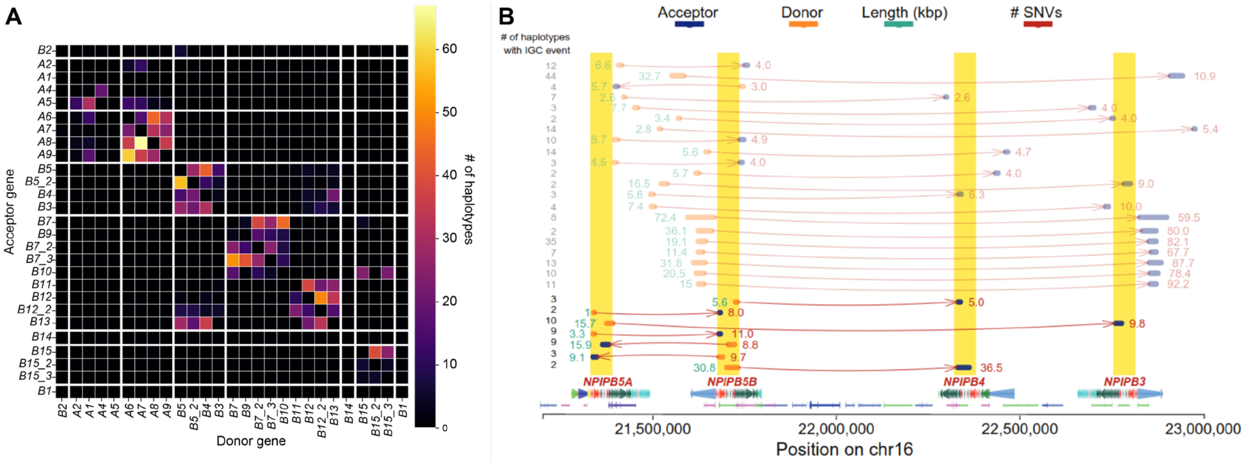

Figure S2. *NPIP* interlocus gene conversion (IGC) and structural changes, related to Figure 3. **A)** Counts of IGC events detected between paralogs for a subset of 94 haplotypes. X-axis labels indicate the IGC donor, and the y-axis denotes the acceptor. **B)** IGC events at the *B3-5* locus. Arrows indicate acceptors (blue) and donors (orange). The length of each event and number of SNVs are shown to the left and right, respectively.

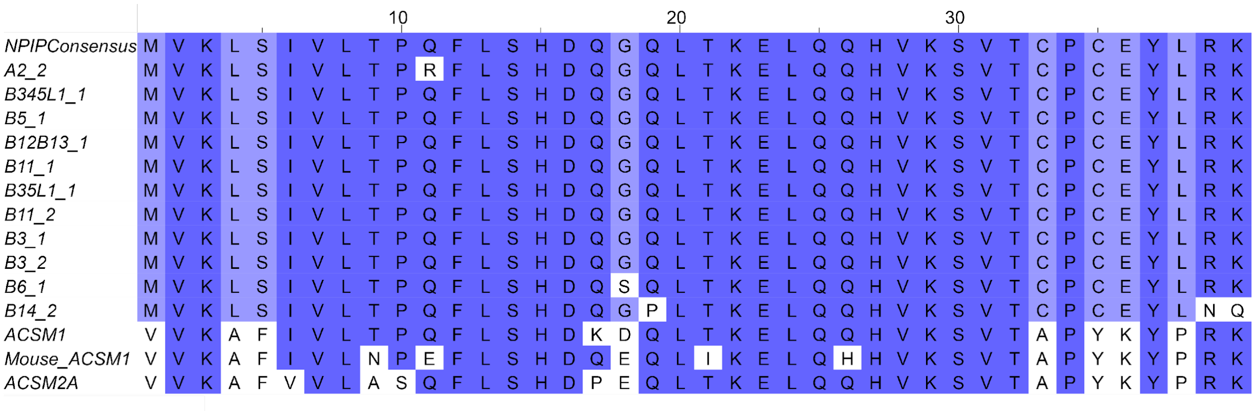

Figure S3. *NPIP* start sequence derived from *ACSM1*, related to Figure 5. Multiple sequence alignment of human *NPIP* paralog start sequences compared to human *ACSM1* and *ACSM2A* and mouse *ACSM1*.

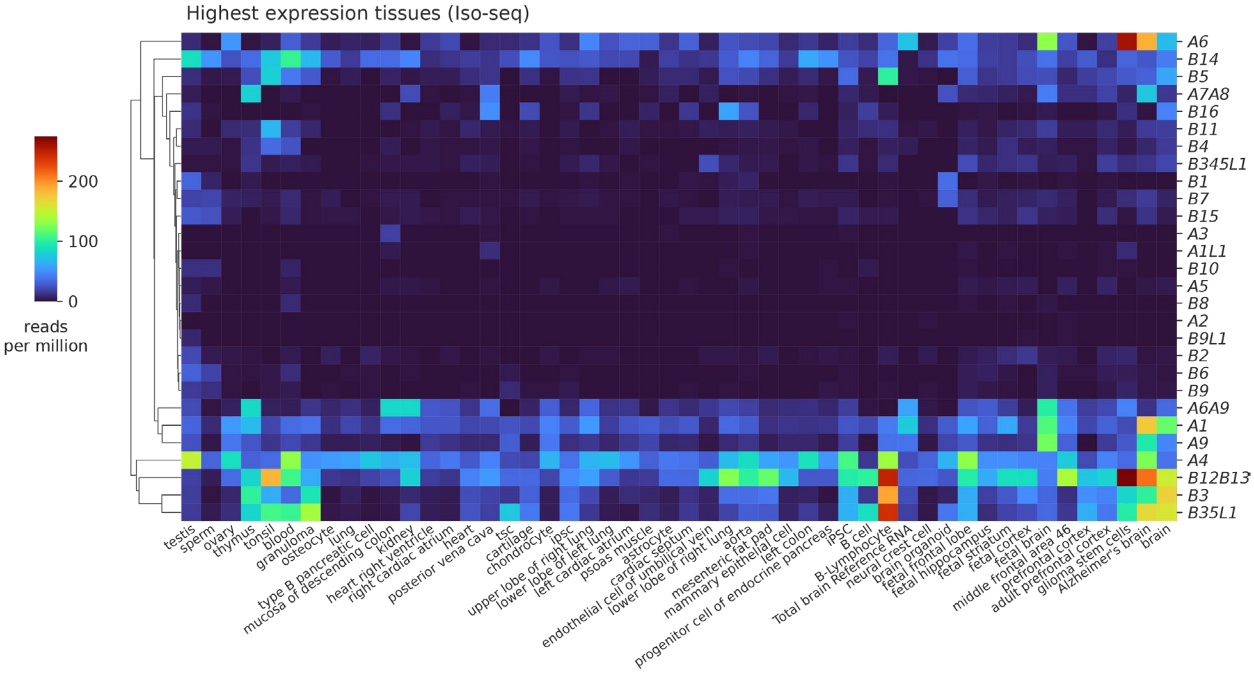

Figure S4. Variable expression of *NPIP* paralogs across tissues and cell types, related to Figure 6. Iso-Seq expression estimates for 50 tissues showing the highest *NPIP* expression, clustered with UPGMA.

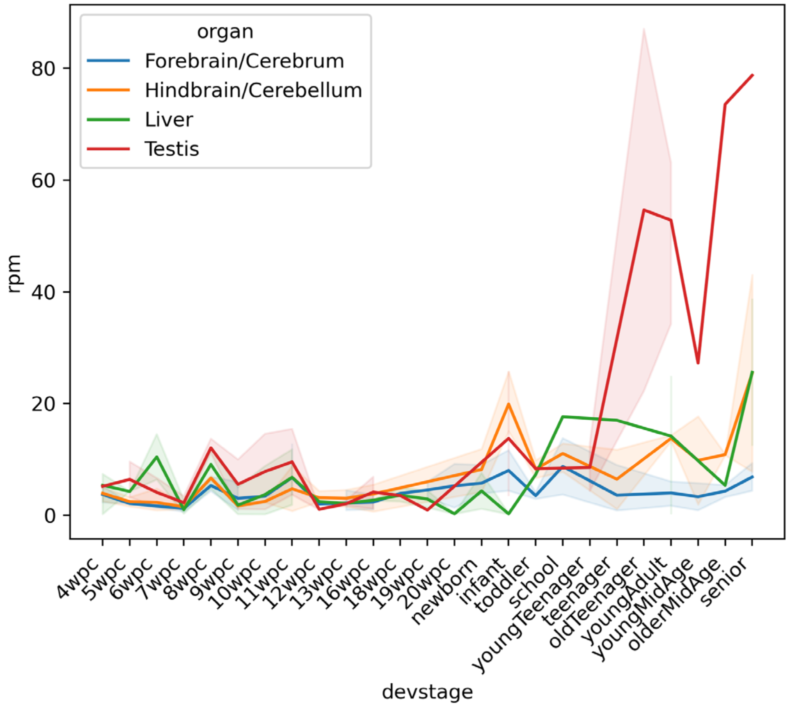

Figure S5. *NPIPB15* expression across development, related to Figure 6. Short-read expression estimates for human developmental timepoints in four tissues, using unique k-mers for paralog identity. Transparent error bands represent 95% confidence interval of replicates.

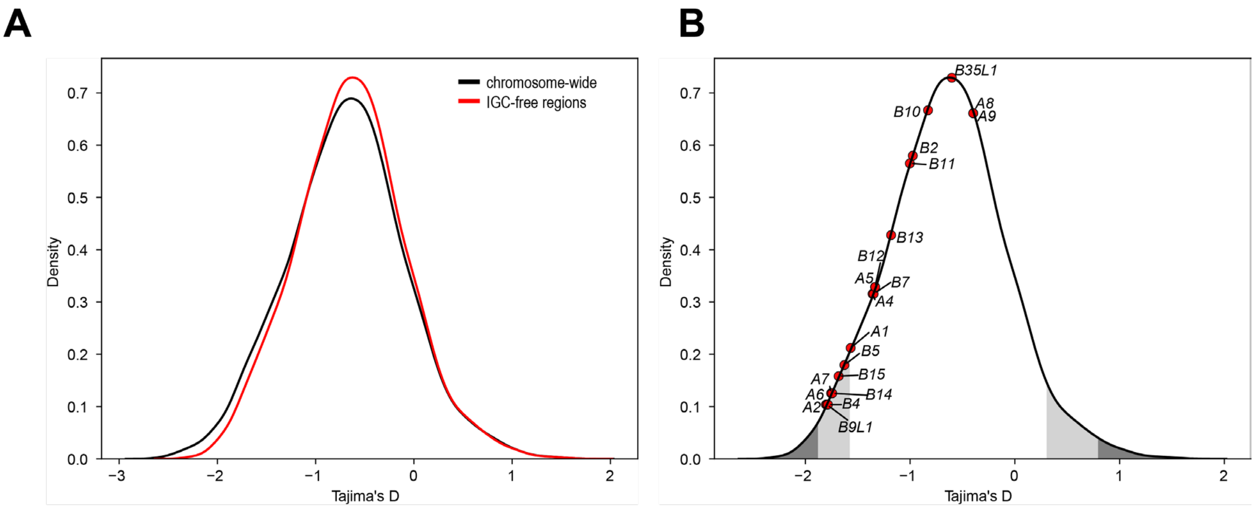

Figure S6. Tajima's D distribution in IGC-free regions, related to Figure 4. A) Tajima's D values for African individuals for the entirety of callable regions on chromosome 16 compared to IGC-free regions of chromosome 16. **B)** Tajima's D values for IGC-free windows nearest to each *NPIP* paralog. The most extreme 1% and 5%, both positive (balancing selection) and negative (positive selection) are colored in gray and dark gray.

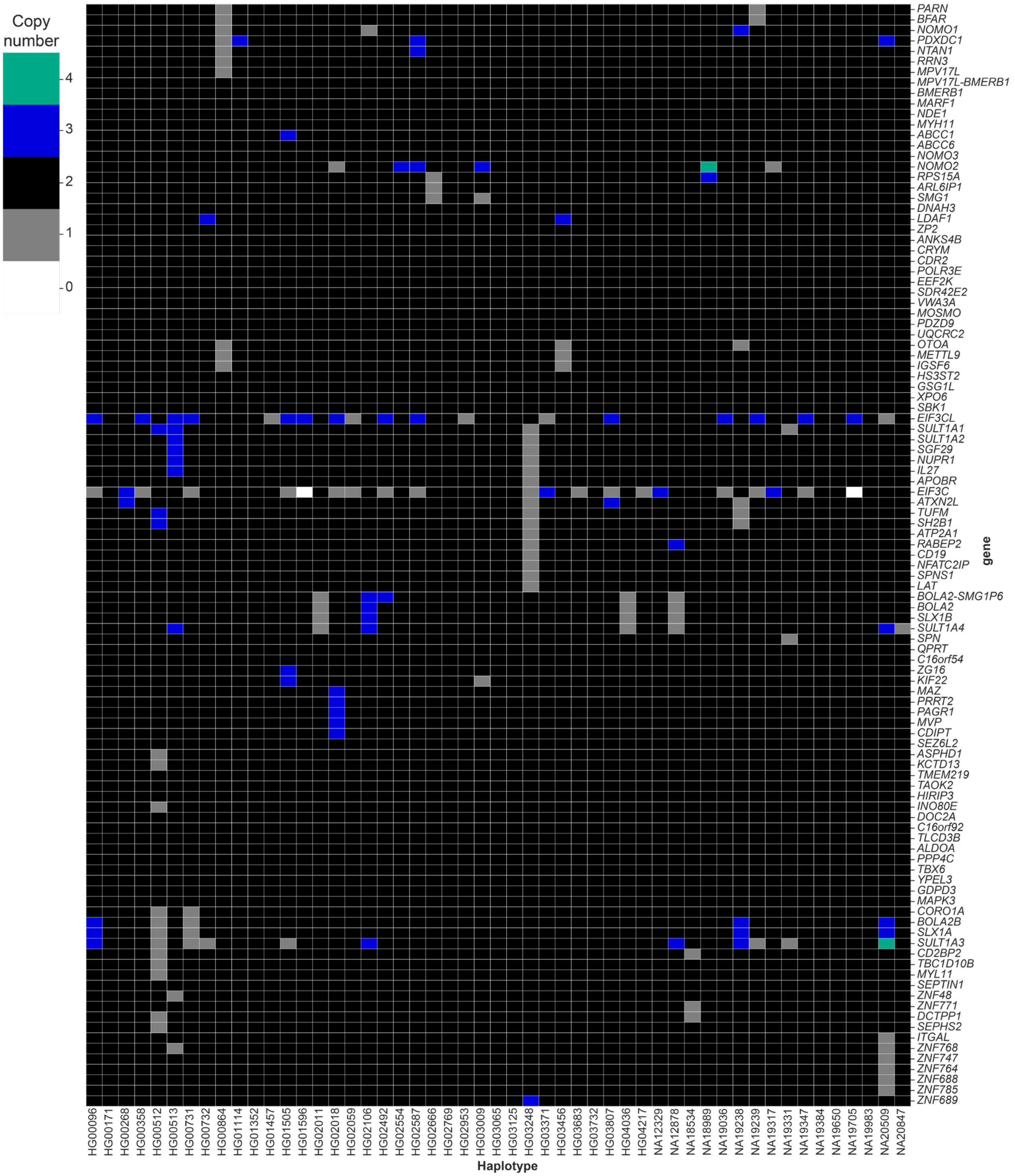

Figure S7. Estimated CN of genes in *NPIP* loci from 102 haplotypes, related to Figure 2. Genes within 30 kbp of an *NPIP* SD block (chr16p13.12-p11.2) are included. Only variable genes shown and 66/105 genes show copy number variation. Rows correspond to genes in chromosome order, and each column represents an assembled sample, with the two haplotypes summed. See Figure 1 for locations of some of the SD genes and Table S4 for detailed breakdown.

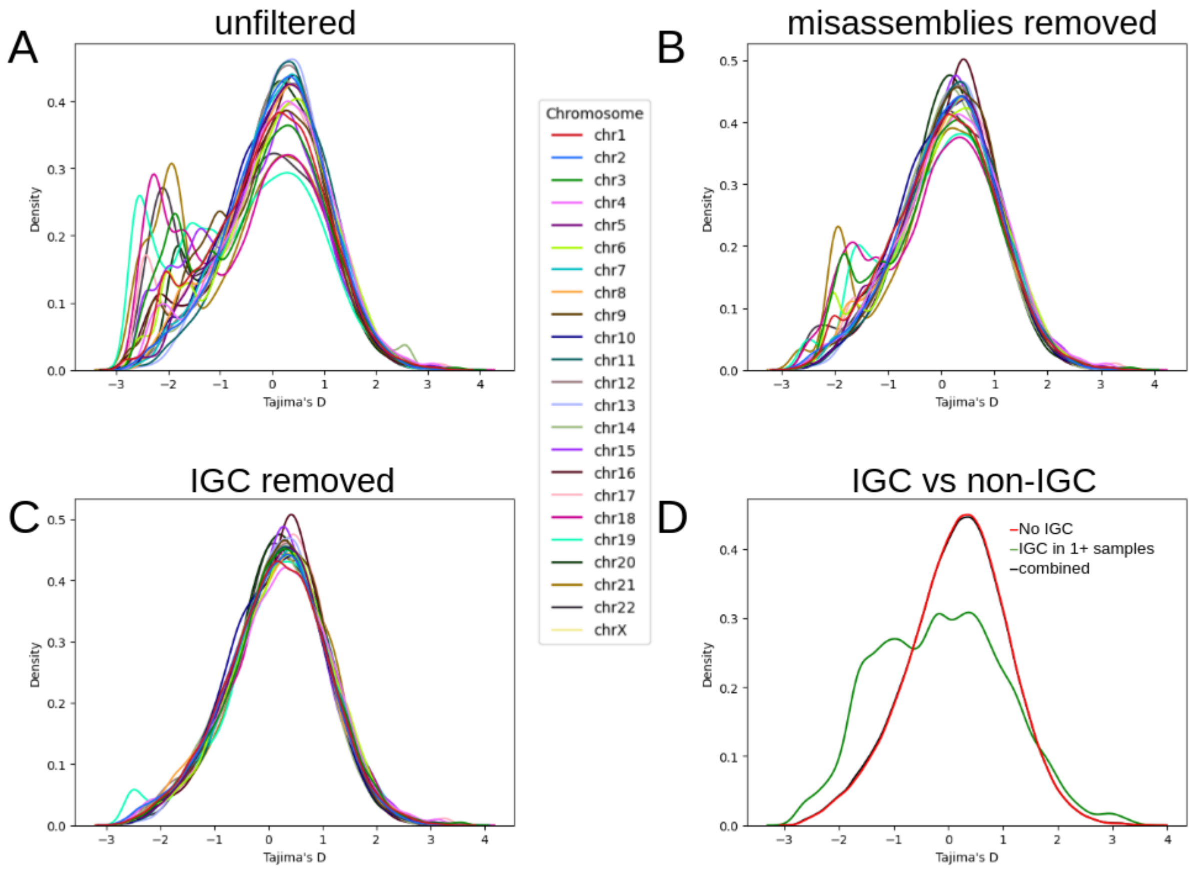

Figure S8. Effect of assembly filtering on Tajima's D distribution, related to Figure 4. A-C) Tajima's D distributions per chromosome from 20 long-read European assemblies (40 haplotypes). **A)** No filtering applied. **B)** Uncallable (ambiguously aligned) regions and misassemblies removed. **C)** IGC tracts removed. **D)** Genome-wide Tajima's D with (black) and without (red) IGC tracts included and limited to only regions of IGC (green).

Table S4. **Copy number of non-NPIP genes within and flanking SD blocks, related to Figure 2.**

Name	Start (T2T chr 16)	End	Min CN	Max CN	Mean	Median	Std Dev
PARN	14473312	14667845	1	2	1.96	2	0.20
BFAR	14670491	14706771	1	2	1.96	2	0.20
NOMO1	14832116	14894560	1	3	1.98	2	0.24
PDXDC1	14975789	15144257	1	3	2.04	2	0.28
NTAN1	15038909	15057126	1	3	2.00	2	0.20
RRN3	15061074	15095386	1	2	1.98	2	0.14
MPV17L	15400550	15418071	1	2	1.98	2	0.14
MPV17L-BMERB1	15400550	15593064	2	2	2.00	2	0.00
BMERB1	15439375	15593064	2	2	2.00	2	0.00
MARF1	15599246	15648059	2	2	2.00	2	0.00
NDE1	15648287	15731290	2	2	2.00	2	0.00
MYH11	15708067	15861962	2	2	2.00	2	0.00
ABCC1	15954068	16147908	2	3	2.02	2	0.14
ABCC6	16154422	16229003	2	2	2.00	2	0.00
NOMO3	16238081	16300368	2	2	2.00	2	0.00
NOMO2	18431048	18493293	1	4	2.06	2	0.42
RPS15A	18717732	18726769	1	3	2.00	2	0.20
ARL6IP1	18728102	18737985	1	2	1.98	2	0.14
SMG1	18741287	18857007	1	2	1.96	2	0.20
DNAH3	20864026	21090383	2	2	2.00	2	0.00
LDAF1	21089598	21111555	2	3	2.04	2	0.20
ZP2	21128399	21145450	2	2	2.00	2	0.00
ANKS4B	21168996	21189143	2	2	2.00	2	0.00
CRYM	21193814	21238355	2	2	2.00	2	0.00
CDR2	21842595	21871275	2	2	2.00	2	0.00
POLR3E	21882112	21919803	2	2	2.00	2	0.00
EEF2K	21928475	22010936	2	2	2.00	2	0.00
SDR42E2	22025463	22054707	2	2	2.00	2	0.00
VWA3A	22060249	22125572	2	2	2.00	2	0.00
MOSMO	22132537	22209081	2	2	2.00	2	0.00
PDZD9	22216077	22259661	2	2	2.00	2	0.00
UQCRC2	22233531	22263842	2	2	2.00	2	0.00
OTOA	22456214	22552969	1	2	1.94	2	0.24
METTL9	22559463	22619737	1	2	1.96	2	0.20
IGSF6	22564326	22577384	1	2	1.96	2	0.20
HS3ST2	23091844	23194123	2	2	2.00	2	0.00
GSG1L	28066522	28344175	2	2	2.00	2	0.00
XPO6	28378417	28492441	2	2	2.00	2	0.00
SBK1	28539191	28604353	2	2	2.00	2	0.00

EIF3CL	28659992	28707115	1	3	2.18	2	0.59
SULT1A1	28772308	28790437	1	3	2.00	2	0.28
SULT1A2	28798642	28803752	1	3	2.00	2	0.20
SGF29	28803905	28857252	1	3	2.00	2	0.20
NUPR1	28856812	28863107	1	3	2.00	2	0.20
IL27	28869090	28896415	1	3	2.00	2	0.20
APOBR	28896813	28901107	1	2	1.98	2	0.14
EIF3C	28969279	29016311	1	3	1.78	2	0.59
ATXN2L	29103578	29117815	1	3	2.00	2	0.28
TUFM	29122980	29140210	1	3	1.98	2	0.24
SH2B1	29127175	29154707	1	3	1.98	2	0.24
ATP2A1	29158988	29184960	1	2	1.98	2	0.14
RABEP2	29184915	29206347	1	3	2.00	2	0.20
CD19	29213087	29220458	1	2	1.98	2	0.14
NFATC2IP	29232053	29248207	1	2	1.98	2	0.14
SPNS1	29255893	29265880	1	2	1.98	2	0.14
LAT	29265918	29271899	1	2	1.98	2	0.14
BOLA2-SMG1P6	29724697	29736755	1	3	1.98	2	0.32
BOLA2	29735379	29736755	1	3	1.96	2	0.28
SLX1B	29736324	29740011	1	3	1.96	2	0.28
SULT1A4	29741703	29746756	1	3	1.98	2	0.37
SPN	29945006	29952917	1	2	1.98	2	0.14
QPRT	29947774	29980720	2	2	2.00	2	0.00
C16orf54	30024601	30028756	2	2	2.00	2	0.00
ZG16	30060382	30065096	2	3	2.02	2	0.14
KIF22	30072908	30087551	1	3	2.00	2	0.20
MAZ	30088290	30093330	2	3	2.02	2	0.14
PRRT2	30094359	30098044	2	3	2.02	2	0.14
PAGR1	30098315	30104650	2	3	2.02	2	0.14
MVP	30102554	30130675	2	3	2.02	2	0.14
CDIPT	30140992	30145861	2	3	2.02	2	0.14
SEZ6L2	30153803	30182186	2	2	2.00	2	0.00
ASPHD1	30183028	30203324	1	2	1.98	2	0.14
KCTD13	30188975	30208863	1	2	1.98	2	0.14
TMEM219	30244712	30255683	2	2	2.00	2	0.00
TAOK2	30256503	30274899	2	2	2.00	2	0.00
HIRIP3	30274959	30278736	2	2	2.00	2	0.00
INO80E	30278881	30288431	1	2	1.98	2	0.14
DOC2A	30288154	30305880	2	2	2.00	2	0.00
C16orf92	30305831	30310369	2	2	2.00	2	0.00
TLCD3B	30307060	30335676	2	2	2.00	2	0.00
ALDOA	30346925	30353065	2	2	2.00	2	0.00
PPP4C	30358641	30368019	2	2	2.00	2	0.00

TBX6	30368436	30374583	2	2	2.00	2	0.00
YPEL3	30374954	30378853	2	2	2.00	2	0.00
GDPD3	30387452	30396184	2	2	2.00	2	0.00
MAPK3	30396752	30405867	2	2	2.00	2	0.00
CORO1A	30468184	30473658	1	2	1.96	2	0.20
BOLA2B	30477482	30478216	1	3	2.02	2	0.32
SLX1A	30478393	30482118	1	3	2.02	2	0.32
SULT1A3	30483809	30488863	1	4	2.00	2	0.53
CD2BP2	30736975	30741510	1	2	1.96	2	0.20
TBC1D10B	30743304	30756697	1	2	1.98	2	0.14
MYL11	30757137	30764194	1	2	1.98	2	0.14
SEPTIN1	30764338	30769019	2	2	2.00	2	0.00
ZNF48	30764515	30786312	1	2	1.98	2	0.14
ZNF771	30793806	30805618	1	2	1.98	2	0.14
DCTPP1	30809819	30816234	1	2	1.96	2	0.20
SEPHS2	30829870	30832113	1	2	1.98	2	0.14
ITGAL	30859706	30910154	1	2	1.98	2	0.14
ZNF768	30910973	30919446	1	2	1.96	2	0.20
ZNF747	30917336	30921826	1	2	1.98	2	0.14
ZNF764	30940731	30945341	1	2	1.98	2	0.14
ZNF688	30956705	30967326	1	2	1.98	2	0.14
ZNF785	30965340	30972758	1	2	1.98	2	0.14
ZNF689	30989540	30997757	2	3	2.02	2	0.14
